# *cyclo*-Tetra­kis(μ-naphthalene-1,8-dicarboxyl­ato)tetra­kis­[diaqua­(2,2′-bipyridine)­manganese(II)] tetra­hydrate

**DOI:** 10.1107/S1600536810049433

**Published:** 2010-11-30

**Authors:** Ling Chen

**Affiliations:** a School of Pharmacy and Material Engineering, Jinhua College of Vocation and Technology, Jinhua, Zhejiang 321017, People’s Republic of China

## Abstract

In the title centrosymmetric tetra­nuclear complex, [Mn_4_(C_12_H_6_O_4_)_4_(C_10_H_8_N_2_)_4_(H_2_O)_8_]·4H_2_O, two independent Mn^II^ ions are coordinated in a slightly disorted octa­hedral environment by two aqua ligands, two naphthalene-1,8-dicarboxyl­ate (1,8-nap) ligands and one bis-chelating 2,2′-bipyridine (2,2′-bipy) ligand. In the crystal, mol­ecules are linked by inter­molecular O—H⋯O hydrogen bonds into chains along [100]. These chains are further linked by weak π–π inter­actions with centroid–centroid distances in the range of 3.609 (2)–3.758 (1) Å, forming a three-dimensional supra­molecular network.

## Related literature

For related structures, see: Feng *et al.* (2008 ▶); Fu *et al.* (2010 ▶); Wen *et al.* (2007 ▶, 2008 ▶).
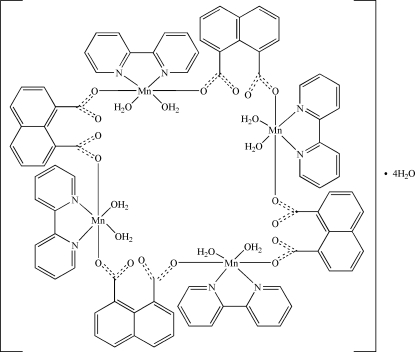


## Experimental

### 

#### Crystal data

[Mn_4_(C_12_H_6_O_4_)_4_(C_10_H_8_N_2_)_4_(H_2_O)_8_]·4H_2_O
*M* *_r_* = 1917.36
Triclinic, 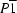

*a* = 10.3323 (3) Å
*b* = 14.3847 (4) Å
*c* = 15.4299 (5) Å
α = 77.760 (2)°
β = 74.198 (2)°
γ = 76.009 (2)°
*V* = 2114.77 (11) Å^3^
*Z* = 1
Mo *K*α radiation
μ = 0.67 mm^−1^
*T* = 296 K
0.37 × 0.17 × 0.05 mm

#### Data collection

Bruker APEXII area-detector diffractometer
Absorption correction: multi-scan (*SADABS*; Sheldrick, 1996 ▶) *T*_min_ = 0.87, *T*_max_ = 0.97
32295 measured reflections
9641 independent reflections
6196 reflections with *I* > 2σ(*I*)
*R*_int_ = 0.041

#### Refinement

*R*[*F* ^2^ > 2σ(*F* ^2^)] = 0.045
*wR*(*F* ^2^) = 0.115
*S* = 1.06
9641 reflections
577 parameters
3 restraints
H-atom parameters constrained
Δρ_max_ = 0.50 e Å^−3^
Δρ_min_ = −0.41 e Å^−3^

### 

Data collection: *APEX2* (Bruker, 2006 ▶); cell refinement: *SAINT* (Bruker, 2006 ▶); data reduction: *SAINT*; program(s) used to solve structure: *SHELXS97* (Sheldrick, 2008 ▶); program(s) used to refine structure: *SHELXL97* (Sheldrick, 2008 ▶); molecular graphics: *DIAMOND* (Brandenburg, 1999) ▶; software used to prepare material for publication: *SHELXTL* (Sheldrick, 2008 ▶).

## Supplementary Material

Crystal structure: contains datablocks I, global. DOI: 10.1107/S1600536810049433/lh5168sup1.cif

Structure factors: contains datablocks I. DOI: 10.1107/S1600536810049433/lh5168Isup2.hkl

Additional supplementary materials:  crystallographic information; 3D view; checkCIF report

## Figures and Tables

**Table 1:** Hydrogen-bond geometry (Å, °)

*D*—H⋯*A*	*D*—H	H⋯*A*	*D*⋯*A*	*D*—H⋯*A*
O1*W*—H1*WA*⋯O2	0.85	2.00	2.690 (2)	139
O1*W*—H1*WB*⋯O6^i^	0.84	2.01	2.776 (2)	151
O2*W*—H2*WA*⋯O4	0.83	1.90	2.712 (2)	166
O2*W*—H2*WB*⋯O5*W*	0.83	1.90	2.716 (3)	169
O3*W*—H3*WA*⋯O8	0.84	1.92	2.742 (2)	167
O3*W*—H3*WB*⋯O2*W*	0.84	2.24	3.083 (3)	180
O4*W*—H4*WA*⋯O6	0.87	1.78	2.622 (2)	161
O4*W*—H4*WB*⋯O6*W*	0.83	2.03	2.853 (3)	170
O5*W*—H5*WA*⋯O7^i^	0.97	2.16	2.807 (3)	123
O5*W*—H5*WB*⋯O6*W*	0.94	2.07	2.930 (3)	150
O6*W*—H6*WA*⋯O7^ii^	0.84	1.99	2.798 (3)	164
O6*W*—H6*WB*⋯O3	0.81	1.95	2.752 (3)	171

Symmetry codes: (i) 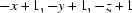; (ii) 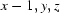.

**Fig. 1.**
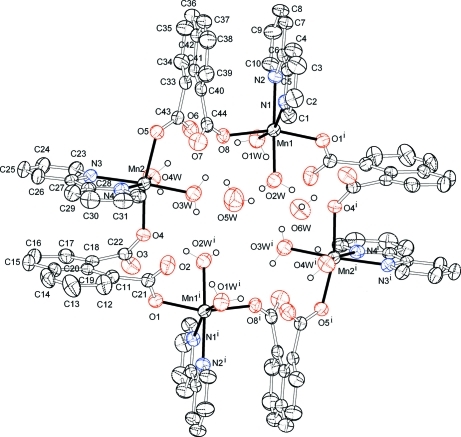
The molecular structure of (I), showing the atom-numbering scheme. Displacement ellipsoids are drawn at the 30% probability level. [Symmetry codes: (i) -x+1, -y+1, -z+1]

**Fig. 2.**
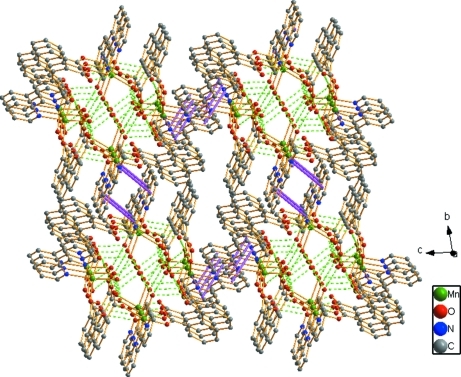
Part of the crystal structure with green dashed lines to show the donor-acceptor distances of hydrogen bonds (H atoms are not shown) and pink lines to show the π–π interactions.

## supplementary crystallographic information

### Comment

Presently, our studies are focused on selecting suitable multidentate ligands to construct novel coordination architectures. 1,8-Naphthalenecarboxylic anhydride, which is can be hydrolysed to naphthalene-1,8-dicarboxylate(1,8-nap) under hydrothermal conditions, is a versatile building block which can be used to construct interesting structures due to the potential variety of bridging abilities. Some complexes containing 1,8-nap have already  been reported (Fu *et al.*, 2010; Feng *et al.*, 2008; Wen *et al.*, 2008; Wen *et al.*, 2007). Herein,we report a new Mn(II) complex containing a 1,8-nap ligand, [Mn_4_(1,8-nap)_4_(2,2'-bipy)_4_(H_2_O)_8_].4(H_2_O),(I).

The molecular structure of (I) is shown in Fig.1. The formula unit consists of four Mn atoms, four 1,8-nap anions, eight coordinated water molecules, two 2,2'-bipy and four lattice water molecules. Unique atoms Mn1 and Mn2 are six-coordinated and have slightly distorted octahedral coordination environments formed by two 1,8-nap ligands, two N atoms from one 2,2'-bipy and two water molecules. All carboxylate groups of the 1,8-nap ligands are deprotonated, and adopt a monodentate coordination mode. Each 1,8-nap ligand links two Mn^II^ ions and hence each Mn^II^ ion coordinates to two 1,8-nap ligands to form a [Mn_4_(1,8-nap)_4_] neutral ring. Four 2,2'-bipy ligands are oriented to the outer side of the ring while the aqua ligands point to the inside of the ring.

In the crystal structure, (Fig. 2) intermolecular O—H···O hydrogen bonds involving coordinated water molecules, solvent water molecules and carboxylate group oxygen atoms, link molecules to form a one-dimensional chain along [100]. In addition, weak π–π interactions with centroid-centroid distance in the range of 3.609 (2) to 3.758 (1) Å between symmetry related 2,2'-bipy ligands  lead to the the formation of a three-dimensional network.

### Experimental

A mixture of MnCl_2_(0.1003 g, 0.5 mmol), naphthalene-1,8-dicarboxylic anhydride (0.0991 g, 0.5 mmol), NaOH (0.0402 g, 1 mmol) and 2,2'-bipyridine (0.0378 g, 0.25 mmol) and ethanol-water (15 ml, V/V, 1:2) was sealed in a 25 ml stainless steel reactor with a Telflon liner and heated at 433 K for 72 h. On completion of the reaction, the reactor was cooled slowly to room temperature and the mixture was filtered, giving colourless block-shaped single crystals suitable for X-ray analysis.

### Refinement

H-atoms were positioned geometrically and included in the refinement using a riding-model approximation [C–H = 0.93 and O–H = 'as found' positions] with *U*_iso_(H) = 1.2*U*_eq_(C) or 1.5*U*_eq_(O).

### Crystal data

**Table:**

[Mn_4_(C_12_H_6_O_4_)_4_(C_10_H_8_N_2_)_4_(H_2_O)_8_]·4H_2_O	*Z* = 1
*M**_r_* = 1917.36	*F*(000) = 988
Triclinic, *P*1	*D*_x_ = 1.506 Mg m^−^^3^
Hall symbol: -P 1	Mo *K*α radiation, λ = 0.71073 Å
*a* = 10.3323 (3) Å	Cell parameters from 5948 reflections
*b* = 14.3847 (4) Å	θ = 1.5–27.6°
*c* = 15.4299 (5) Å	µ = 0.67 mm^−^^1^
α = 77.760 (2)°	*T* = 296 K
β = 74.198 (2)°	Block, colourless
γ = 76.009 (2)°	0.37 × 0.17 × 0.05 mm
*V* = 2114.77 (11) Å^3^	

### Data collection

**Table:**

Bruker APEXII area-detector diffractometer	9641 independent reflections
Radiation source: fine-focus sealed tube	6196 reflections with *I* > 2σ(*I*)
graphite	*R*_int_ = 0.041
ω scans	θ_max_ = 27.6°, θ_min_ = 1.5°
Absorption correction: multi-scan (*SADABS*; Sheldrick, 1996)	*h* = −12→13
*T*_min_ = 0.87, *T*_max_ = 0.97	*k* = −18→18
32295 measured reflections	*l* = −19→20

### Refinement

**Table:**

Refinement on *F*^2^	Primary atom site location: structure-invariant direct methods
Least-squares matrix: full	Secondary atom site location: difference Fourier map
*R*[*F*^2^ > 2σ(*F*^2^)] = 0.045	Hydrogen site location: inferred from neighbouring sites
*wR*(*F*^2^) = 0.115	H-atom parameters constrained
*S* = 1.06	*w* = 1/[σ^2^(*F*_o_^2^) + (0.0516*P*)^2^] where *P* = (*F*_o_^2^ + 2*F*_c_^2^)/3
9641 reflections	(Δ/σ)_max_ = 0.001
577 parameters	Δρ_max_ = 0.50 e Å^−^^3^
3 restraints	Δρ_min_ = −0.41 e Å^−^^3^

### Special details

**Table:**

Geometry. All e.s.d.'s (except the e.s.d. in the dihedral angle between two l.s. planes) are estimated using the full covariance matrix. The cell e.s.d.'s are taken into account individually in the estimation of e.s.d.'s in distances, angles and torsion angles; correlations between e.s.d.'s in cell parameters are only used when they are defined by crystal symmetry. An approximate (isotropic) treatment of cell e.s.d.'s is used for estimating e.s.d.'s involving l.s. planes.
Refinement. Refinement of *F*^2^ against ALL reflections. The weighted *R*-factor *wR* and goodness of fit *S* are based on *F*^2^, conventional *R*-factors *R* are based on *F*, with *F* set to zero for negative *F*^2^. The threshold expression of *F*^2^ > σ(*F*^2^) is used only for calculating *R*-factors(gt) *etc*. and is not relevant to the choice of reflections for refinement. *R*-factors based on *F*^2^ are statistically about twice as large as those based on *F*, and *R*- factors based on ALL data will be even larger.

### Fractional atomic coordinates and isotropic or equivalent isotropic displacement parameters (Å^2^)

**Table:**

	*x*	*y*	*z*	*U*_iso_*/*U*_eq_	
Mn1	0.25463 (4)	0.76117 (3)	0.46946 (2)	0.03213 (11)	
Mn2	0.38953 (4)	0.49105 (3)	0.78495 (2)	0.03230 (12)	
O1	0.25004 (16)	0.79966 (12)	0.59713 (10)	0.0354 (4)	
O1W	0.46961 (17)	0.77539 (13)	0.42064 (12)	0.0494 (5)	
H1WA	0.5029	0.7430	0.4648	0.074*	
H1WB	0.5219	0.7510	0.3756	0.074*	
O2	0.46969 (17)	0.73941 (15)	0.59898 (12)	0.0540 (5)	
O2W	0.2899 (2)	0.61536 (13)	0.54280 (11)	0.0602 (6)	
H2WA	0.2749	0.6061	0.5991	0.090*	
H2WB	0.2769	0.5661	0.5290	0.090*	
O3	0.03335 (18)	0.65873 (13)	0.73071 (13)	0.0510 (5)	
O3W	0.50323 (18)	0.48414 (14)	0.64458 (11)	0.0562 (5)	
H3WA	0.5675	0.4364	0.6344	0.084*	
H3WB	0.4454	0.5200	0.6167	0.084*	
O4	0.25706 (16)	0.61265 (11)	0.72371 (10)	0.0354 (4)	
O4W	0.22730 (17)	0.40881 (13)	0.79531 (12)	0.0482 (5)	
H4WA	0.2859	0.3596	0.7735	0.072*	
H4WB	0.1749	0.4249	0.7597	0.072*	
O5	0.52168 (16)	0.35117 (12)	0.81890 (11)	0.0377 (4)	
O5W	0.2207 (3)	0.47052 (17)	0.48764 (16)	0.0944 (8)	
H5WA	0.1509	0.4921	0.4530	0.142*	
H5WB	0.1449	0.4691	0.5379	0.142*	
O6	0.43026 (18)	0.26289 (13)	0.75699 (12)	0.0477 (5)	
O6W	0.05204 (19)	0.48703 (14)	0.67218 (14)	0.0626 (6)	
H6WA	−0.0250	0.4730	0.6810	0.094*	
H6WB	0.0380	0.5395	0.6880	0.094*	
O7	0.82217 (19)	0.41392 (14)	0.67703 (13)	0.0521 (5)	
O8	0.70690 (16)	0.32143 (12)	0.64178 (10)	0.0357 (4)	
N1	0.0249 (2)	0.79384 (15)	0.47871 (13)	0.0358 (5)	
N2	0.1971 (2)	0.91457 (14)	0.39807 (13)	0.0344 (5)	
N3	0.2996 (2)	0.52060 (14)	0.92913 (12)	0.0346 (5)	
N4	0.5154 (2)	0.58391 (14)	0.81433 (13)	0.0359 (5)	
C1	−0.0580 (3)	0.7300 (2)	0.51645 (18)	0.0473 (7)	
H1A	−0.0206	0.6685	0.5426	0.057*	
C2	−0.1956 (3)	0.7512 (2)	0.5184 (2)	0.0566 (8)	
H2A	−0.2496	0.7047	0.5448	0.068*	
C3	−0.2516 (3)	0.8414 (2)	0.4810 (2)	0.0588 (8)	
H3A	−0.3448	0.8577	0.4823	0.071*	
C4	−0.1689 (3)	0.9084 (2)	0.44113 (19)	0.0496 (7)	
H4A	−0.2054	0.9702	0.4149	0.060*	
C5	−0.0304 (2)	0.88263 (19)	0.44059 (15)	0.0341 (6)	
C6	0.0646 (2)	0.95046 (17)	0.39762 (15)	0.0311 (6)	
C7	0.0205 (3)	1.04443 (19)	0.35954 (17)	0.0430 (7)	
H7A	−0.0720	1.0676	0.3605	0.052*	
C8	0.1140 (3)	1.1040 (2)	0.31990 (18)	0.0475 (7)	
H8A	0.0857	1.1677	0.2938	0.057*	
C9	0.2486 (3)	1.0680 (2)	0.31958 (19)	0.0505 (7)	
H9A	0.3139	1.1065	0.2932	0.061*	
C10	0.2862 (3)	0.97346 (19)	0.35893 (18)	0.0446 (7)	
H10A	0.3784	0.9493	0.3583	0.053*	
C11	0.3276 (2)	0.81466 (19)	0.72314 (16)	0.0370 (6)	
C12	0.4079 (3)	0.8776 (2)	0.7214 (2)	0.0562 (8)	
H12A	0.4758	0.8895	0.6690	0.067*	
C13	0.3931 (3)	0.9251 (3)	0.7948 (2)	0.0705 (10)	
H13A	0.4484	0.9690	0.7905	0.085*	
C14	0.2979 (3)	0.9066 (2)	0.8714 (2)	0.0630 (9)	
H14A	0.2889	0.9370	0.9208	0.076*	
C15	0.1091 (3)	0.8272 (2)	0.95932 (19)	0.0625 (9)	
H15A	0.1045	0.8563	1.0088	0.075*	
C16	0.0179 (4)	0.7714 (2)	0.96595 (19)	0.0650 (9)	
H16A	−0.0509	0.7641	1.0188	0.078*	
C17	0.0273 (3)	0.7245 (2)	0.89283 (18)	0.0513 (7)	
H17A	−0.0370	0.6873	0.8972	0.062*	
C18	0.1297 (3)	0.73254 (18)	0.81485 (16)	0.0372 (6)	
C19	0.2234 (2)	0.79467 (18)	0.80346 (16)	0.0359 (6)	
C20	0.2114 (3)	0.8423 (2)	0.87871 (17)	0.0476 (7)	
C21	0.3518 (3)	0.77905 (18)	0.63364 (16)	0.0344 (6)	
C22	0.1399 (3)	0.66459 (18)	0.75014 (16)	0.0352 (6)	
C23	0.1922 (3)	0.48646 (19)	0.98448 (16)	0.0415 (6)	
H23A	0.1534	0.4463	0.9633	0.050*	
C24	0.1356 (3)	0.5076 (2)	1.07131 (17)	0.0500 (7)	
H24A	0.0600	0.4828	1.1077	0.060*	
C25	0.1930 (3)	0.5658 (2)	1.10280 (17)	0.0508 (7)	
H25A	0.1575	0.5810	1.1614	0.061*	
C26	0.3036 (3)	0.60142 (19)	1.04707 (17)	0.0457 (7)	
H26A	0.3440	0.6409	1.0678	0.055*	
C27	0.3552 (2)	0.57877 (17)	0.95985 (16)	0.0339 (6)	
C28	0.4746 (2)	0.61537 (18)	0.89526 (16)	0.0353 (6)	
C29	0.5408 (3)	0.6765 (2)	0.91684 (19)	0.0502 (7)	
H29A	0.5103	0.6979	0.9731	0.060*	
C30	0.6526 (3)	0.7058 (2)	0.8545 (2)	0.0595 (8)	
H30A	0.6986	0.7470	0.8681	0.071*	
C31	0.6950 (3)	0.6735 (2)	0.7725 (2)	0.0539 (8)	
H31A	0.7709	0.6918	0.7296	0.065*	
C32	0.6240 (3)	0.61362 (19)	0.75418 (19)	0.0444 (7)	
H32A	0.6524	0.5927	0.6977	0.053*	
C33	0.6221 (3)	0.18375 (19)	0.81790 (16)	0.0399 (6)	
C34	0.5646 (3)	0.1055 (2)	0.86429 (19)	0.0566 (8)	
H34A	0.4707	0.1104	0.8730	0.068*	
C35	0.6422 (4)	0.0185 (2)	0.8990 (2)	0.0677 (9)	
H35A	0.6001	−0.0330	0.9308	0.081*	
C36	0.7771 (4)	0.0106 (2)	0.8860 (2)	0.0683 (10)	
H36A	0.8284	−0.0473	0.9091	0.082*	
C37	0.9870 (4)	0.0775 (3)	0.8269 (2)	0.0681 (10)	
H37A	1.0369	0.0190	0.8501	0.082*	
C38	1.0529 (3)	0.1503 (3)	0.7835 (2)	0.0718 (10)	
H38A	1.1460	0.1436	0.7798	0.086*	
C39	0.9791 (3)	0.2366 (2)	0.74377 (19)	0.0549 (8)	
H39A	1.0251	0.2866	0.7132	0.066*	
C40	0.8424 (3)	0.2495 (2)	0.74856 (16)	0.0406 (6)	
C41	0.7661 (3)	0.17662 (19)	0.80045 (16)	0.0416 (7)	
C42	0.8443 (3)	0.0873 (2)	0.83827 (18)	0.0523 (8)	
C43	0.5196 (3)	0.27347 (19)	0.79374 (16)	0.0364 (6)	
C44	0.7856 (2)	0.3351 (2)	0.68661 (16)	0.0380 (6)	

### Atomic displacement parameters (Å^2^)

**Table:**

	*U*^11^	*U*^22^	*U*^33^	*U*^12^	*U*^13^	*U*^23^
Mn1	0.0333 (2)	0.0320 (2)	0.0315 (2)	−0.00393 (17)	−0.00973 (16)	−0.00581 (16)
Mn2	0.0315 (2)	0.0347 (2)	0.0302 (2)	−0.00291 (17)	−0.00903 (16)	−0.00567 (16)
O1	0.0309 (9)	0.0412 (11)	0.0367 (9)	−0.0018 (8)	−0.0149 (8)	−0.0085 (8)
O1W	0.0375 (11)	0.0660 (14)	0.0461 (11)	−0.0069 (10)	−0.0108 (8)	−0.0143 (10)
O2	0.0279 (10)	0.0872 (16)	0.0441 (10)	0.0038 (10)	−0.0082 (8)	−0.0219 (10)
O2W	0.1071 (17)	0.0392 (12)	0.0356 (10)	−0.0111 (11)	−0.0227 (11)	−0.0040 (9)
O3	0.0370 (11)	0.0511 (13)	0.0691 (13)	−0.0090 (9)	−0.0165 (9)	−0.0124 (10)
O3W	0.0442 (11)	0.0711 (14)	0.0375 (10)	0.0161 (10)	−0.0086 (8)	−0.0071 (10)
O4	0.0353 (9)	0.0334 (10)	0.0339 (9)	0.0017 (7)	−0.0099 (7)	−0.0045 (7)
O4W	0.0425 (11)	0.0464 (12)	0.0609 (12)	−0.0088 (8)	−0.0163 (9)	−0.0144 (9)
O5	0.0408 (10)	0.0348 (10)	0.0376 (9)	0.0003 (8)	−0.0130 (8)	−0.0099 (8)
O5W	0.135 (2)	0.0717 (17)	0.0772 (16)	−0.0367 (16)	−0.0068 (15)	−0.0169 (13)
O6	0.0444 (11)	0.0498 (12)	0.0548 (11)	−0.0058 (9)	−0.0165 (9)	−0.0186 (9)
O6W	0.0503 (12)	0.0620 (14)	0.0833 (15)	−0.0197 (11)	−0.0154 (11)	−0.0184 (12)
O7	0.0491 (12)	0.0523 (13)	0.0646 (13)	−0.0152 (10)	−0.0221 (10)	−0.0123 (10)
O8	0.0347 (10)	0.0420 (11)	0.0327 (9)	−0.0029 (8)	−0.0115 (7)	−0.0117 (8)
N1	0.0365 (12)	0.0349 (13)	0.0365 (11)	−0.0080 (10)	−0.0066 (9)	−0.0080 (10)
N2	0.0313 (12)	0.0341 (12)	0.0376 (11)	−0.0053 (10)	−0.0096 (9)	−0.0049 (9)
N3	0.0363 (12)	0.0364 (12)	0.0306 (11)	−0.0052 (10)	−0.0102 (9)	−0.0036 (9)
N4	0.0314 (12)	0.0363 (13)	0.0374 (11)	−0.0041 (10)	−0.0093 (9)	−0.0016 (10)
C1	0.0496 (18)	0.0409 (17)	0.0514 (16)	−0.0159 (14)	−0.0083 (13)	−0.0036 (13)
C2	0.0457 (19)	0.062 (2)	0.065 (2)	−0.0248 (17)	−0.0051 (15)	−0.0090 (17)
C3	0.0321 (16)	0.068 (2)	0.076 (2)	−0.0112 (16)	−0.0107 (15)	−0.0121 (18)
C4	0.0324 (16)	0.0529 (19)	0.0619 (18)	−0.0046 (14)	−0.0133 (13)	−0.0071 (15)
C5	0.0307 (14)	0.0386 (16)	0.0334 (13)	−0.0029 (12)	−0.0065 (11)	−0.0123 (12)
C6	0.0339 (14)	0.0308 (15)	0.0286 (12)	−0.0035 (12)	−0.0060 (10)	−0.0098 (11)
C7	0.0376 (15)	0.0410 (17)	0.0476 (16)	0.0038 (13)	−0.0140 (12)	−0.0088 (13)
C8	0.0563 (19)	0.0295 (16)	0.0531 (17)	−0.0040 (14)	−0.0163 (14)	0.0007 (13)
C9	0.0524 (19)	0.0409 (18)	0.0580 (18)	−0.0169 (15)	−0.0138 (14)	0.0028 (14)
C10	0.0364 (15)	0.0404 (17)	0.0557 (17)	−0.0079 (13)	−0.0124 (13)	−0.0027 (14)
C11	0.0325 (14)	0.0418 (16)	0.0407 (14)	−0.0018 (12)	−0.0164 (11)	−0.0110 (12)
C12	0.0413 (17)	0.075 (2)	0.0647 (19)	−0.0169 (16)	−0.0157 (14)	−0.0275 (17)
C13	0.055 (2)	0.081 (3)	0.094 (3)	−0.0157 (19)	−0.0222 (19)	−0.044 (2)
C14	0.058 (2)	0.075 (2)	0.071 (2)	0.0076 (15)	−0.0334 (18)	−0.0450 (19)
C15	0.080 (2)	0.064 (2)	0.0386 (16)	0.0142 (19)	−0.0195 (16)	−0.0222 (16)
C16	0.076 (2)	0.061 (2)	0.0347 (16)	0.0105 (19)	0.0034 (15)	−0.0059 (15)
C17	0.0469 (17)	0.0460 (18)	0.0471 (16)	−0.0014 (14)	0.0010 (13)	−0.0021 (14)
C18	0.0395 (15)	0.0308 (15)	0.0345 (13)	0.0057 (12)	−0.0110 (12)	−0.0018 (11)
C19	0.0346 (14)	0.0356 (15)	0.0365 (13)	0.0070 (12)	−0.0161 (11)	−0.0091 (11)
C20	0.0505 (17)	0.0488 (17)	0.0422 (15)	0.0132 (12)	−0.0207 (13)	−0.0177 (14)
C21	0.0305 (15)	0.0389 (15)	0.0355 (13)	−0.0080 (12)	−0.0089 (11)	−0.0060 (12)
C22	0.0330 (12)	0.0320 (15)	0.0359 (13)	−0.0037 (10)	−0.0074 (11)	0.0010 (11)
C23	0.0430 (16)	0.0453 (17)	0.0382 (14)	−0.0153 (13)	−0.0091 (12)	−0.0037 (12)
C24	0.0515 (18)	0.0550 (19)	0.0380 (15)	−0.0127 (15)	−0.0015 (13)	−0.0041 (14)
C25	0.0591 (19)	0.058 (2)	0.0301 (14)	−0.0049 (16)	−0.0044 (13)	−0.0104 (13)
C26	0.0567 (18)	0.0458 (18)	0.0395 (15)	−0.0067 (15)	−0.0176 (14)	−0.0130 (13)
C27	0.0383 (14)	0.0308 (14)	0.0334 (13)	−0.0007 (12)	−0.0165 (11)	−0.0028 (11)
C28	0.0347 (14)	0.0318 (14)	0.0399 (14)	−0.0007 (12)	−0.0165 (11)	−0.0033 (12)
C29	0.0487 (18)	0.0542 (19)	0.0550 (17)	−0.0127 (15)	−0.0199 (14)	−0.0109 (15)
C30	0.058 (2)	0.055 (2)	0.079 (2)	−0.0227 (17)	−0.0274 (17)	−0.0102 (17)
C31	0.0350 (16)	0.0527 (19)	0.070 (2)	−0.0142 (15)	−0.0120 (14)	0.0043 (16)
C32	0.0336 (15)	0.0435 (17)	0.0510 (16)	−0.0032 (13)	−0.0096 (13)	−0.0022 (13)
C33	0.0509 (18)	0.0347 (16)	0.0338 (13)	0.0002 (13)	−0.0129 (12)	−0.0107 (12)
C34	0.071 (2)	0.0453 (19)	0.0509 (17)	−0.0074 (17)	−0.0137 (15)	−0.0077 (15)
C35	0.099 (3)	0.039 (2)	0.058 (2)	−0.007 (2)	−0.018 (2)	−0.0017 (15)
C36	0.100 (3)	0.045 (2)	0.0519 (19)	0.018 (2)	−0.031 (2)	−0.0111 (16)
C37	0.074 (3)	0.066 (2)	0.054 (2)	0.033 (2)	−0.0330 (18)	−0.0194 (18)
C38	0.052 (2)	0.096 (3)	0.064 (2)	0.020 (2)	−0.0286 (17)	−0.026 (2)
C39	0.0449 (18)	0.068 (2)	0.0556 (18)	0.0027 (16)	−0.0219 (14)	−0.0202 (16)
C40	0.0414 (16)	0.0465 (17)	0.0355 (14)	0.0057 (13)	−0.0148 (12)	−0.0186 (13)
C41	0.0542 (18)	0.0390 (16)	0.0328 (13)	0.0074 (14)	−0.0192 (12)	−0.0153 (12)
C42	0.068 (2)	0.0467 (19)	0.0381 (15)	0.0158 (16)	−0.0225 (14)	−0.0162 (14)
C43	0.0402 (16)	0.0362 (16)	0.0301 (13)	−0.0055 (13)	−0.0050 (11)	−0.0057 (12)
C44	0.0320 (15)	0.0464 (18)	0.0366 (14)	−0.0011 (13)	−0.0074 (11)	−0.0171 (13)

### Geometric parameters (Å, °)

**Table:**

Mn1—O1	2.1422 (15)	C10—H10A	0.9300
Mn1—O2W	2.1590 (18)	C11—C12	1.359 (3)
Mn1—O8^i^	2.1864 (15)	C11—C19	1.432 (3)
Mn1—O1W	2.1878 (17)	C11—C21	1.511 (3)
Mn1—N2	2.264 (2)	C12—C13	1.400 (4)
Mn1—N1	2.276 (2)	C12—H12A	0.9300
Mn2—O4	2.1614 (16)	C13—C14	1.340 (4)
Mn2—O3W	2.1749 (16)	C13—H13A	0.9300
Mn2—O5	2.1913 (17)	C14—C20	1.402 (4)
Mn2—O4W	2.2288 (16)	C14—H14A	0.9300
Mn2—N3	2.2533 (18)	C15—C16	1.350 (4)
Mn2—N4	2.258 (2)	C15—C20	1.411 (4)
O1—C21	1.269 (3)	C15—H15A	0.9300
O1W—H1WA	0.8451	C16—C17	1.403 (4)
O1W—H1WB	0.8403	C16—H16A	0.9300
O2—C21	1.238 (3)	C17—C18	1.374 (3)
O2W—H2WA	0.8269	C17—H17A	0.9300
O2W—H2WB	0.8319	C18—C19	1.423 (3)
O3—C22	1.243 (3)	C18—C22	1.507 (3)
O3W—H3WA	0.8400	C19—C20	1.432 (3)
O3W—H3WB	0.8399	C23—C24	1.375 (3)
O4—C22	1.270 (3)	C23—H23A	0.9300
O4W—H4WA	0.8733	C24—C25	1.363 (4)
O4W—H4WB	0.8342	C24—H24A	0.9300
O5—C43	1.265 (3)	C25—C26	1.367 (4)
O5W—H5WA	0.9661	C25—H25A	0.9300
O5W—H5WB	0.9411	C26—C27	1.383 (3)
O6—C43	1.260 (3)	C26—H26A	0.9300
O6W—H6WA	0.8364	C27—C28	1.488 (3)
O6W—H6WB	0.8096	C28—C29	1.375 (3)
O7—C44	1.247 (3)	C29—C30	1.377 (4)
O8—C44	1.273 (3)	C29—H29A	0.9300
O8—Mn1^i^	2.1864 (15)	C30—C31	1.362 (4)
N1—C1	1.341 (3)	C30—H30A	0.9300
N1—C5	1.347 (3)	C31—C32	1.372 (4)
N2—C10	1.337 (3)	C31—H31A	0.9300
N2—C6	1.342 (3)	C32—H32A	0.9300
N3—C23	1.332 (3)	C33—C34	1.373 (4)
N3—C27	1.343 (3)	C33—C41	1.420 (4)
N4—C32	1.342 (3)	C33—C43	1.509 (3)
N4—C28	1.342 (3)	C34—C35	1.399 (4)
C1—C2	1.374 (4)	C34—H34A	0.9300
C1—H1A	0.9300	C35—C36	1.333 (4)
C2—C3	1.361 (4)	C35—H35A	0.9300
C2—H2A	0.9300	C36—C42	1.411 (4)
C3—C4	1.378 (4)	C36—H36A	0.9300
C3—H3A	0.9300	C37—C38	1.344 (5)
C4—C5	1.386 (3)	C37—C42	1.412 (4)
C4—H4A	0.9300	C37—H37A	0.9300
C5—C6	1.480 (3)	C38—C39	1.405 (4)
C6—C7	1.375 (3)	C38—H38A	0.9300
C7—C8	1.378 (4)	C39—C40	1.363 (4)
C7—H7A	0.9300	C39—H39A	0.9300
C8—C9	1.360 (4)	C40—C41	1.436 (4)
C8—H8A	0.9300	C40—C44	1.494 (4)
C9—C10	1.375 (4)	C41—C42	1.443 (4)
C9—H9A	0.9300		
			
O1—Mn1—O2W	83.03 (6)	C14—C13—C12	119.0 (3)
O1—Mn1—O8^i^	161.96 (6)	C14—C13—H13A	120.5
O2W—Mn1—O8^i^	80.01 (6)	C12—C13—H13A	120.5
O1—Mn1—O1W	89.79 (6)	C13—C14—C20	121.4 (3)
O2W—Mn1—O1W	97.14 (7)	C13—C14—H14A	119.3
O8^i^—Mn1—O1W	86.29 (6)	C20—C14—H14A	119.3
O1—Mn1—N2	96.36 (7)	C16—C15—C20	121.2 (3)
O2W—Mn1—N2	174.79 (7)	C16—C15—H15A	119.4
O8^i^—Mn1—N2	101.08 (6)	C20—C15—H15A	119.4
O1W—Mn1—N2	88.02 (7)	C15—C16—C17	119.7 (3)
O1—Mn1—N1	98.63 (6)	C15—C16—H16A	120.1
O2W—Mn1—N1	103.07 (8)	C17—C16—H16A	120.1
O8^i^—Mn1—N1	91.17 (6)	C18—C17—C16	121.3 (3)
O1W—Mn1—N1	158.87 (8)	C18—C17—H17A	119.4
N2—Mn1—N1	71.87 (7)	C16—C17—H17A	119.4
O4—Mn2—O3W	83.70 (6)	C17—C18—C19	120.6 (2)
O4—Mn2—O5	165.69 (6)	C17—C18—C22	114.4 (2)
O3W—Mn2—O5	85.90 (6)	C19—C18—C22	124.8 (2)
O4—Mn2—O4W	84.94 (6)	C18—C19—C11	125.8 (2)
O3W—Mn2—O4W	100.87 (7)	C18—C19—C20	117.1 (2)
O5—Mn2—O4W	87.41 (6)	C11—C19—C20	117.1 (2)
O4—Mn2—N3	95.56 (6)	C14—C20—C15	119.8 (3)
O3W—Mn2—N3	167.88 (7)	C14—C20—C19	120.2 (3)
O5—Mn2—N3	96.66 (7)	C15—C20—C19	119.9 (3)
O4W—Mn2—N3	91.10 (7)	O2—C21—O1	125.6 (2)
O4—Mn2—N4	94.31 (7)	O2—C21—C11	118.1 (2)
O3W—Mn2—N4	95.66 (7)	O1—C21—C11	116.1 (2)
O5—Mn2—N4	96.47 (7)	O3—C22—O4	124.5 (2)
O4W—Mn2—N4	163.26 (7)	O3—C22—C18	118.5 (2)
N3—Mn2—N4	72.30 (7)	O4—C22—C18	116.8 (2)
C21—O1—Mn1	124.46 (15)	N3—C23—C24	123.3 (2)
Mn1—O1W—H1WA	101.5	N3—C23—H23A	118.3
Mn1—O1W—H1WB	122.4	C24—C23—H23A	118.3
H1WA—O1W—H1WB	103.1	C25—C24—C23	118.4 (3)
Mn1—O2W—H2WA	120.1	C25—C24—H24A	120.8
Mn1—O2W—H2WB	126.4	C23—C24—H24A	120.8
H2WA—O2W—H2WB	105.6	C24—C25—C26	119.1 (2)
Mn2—O3W—H3WA	117.9	C24—C25—H25A	120.4
Mn2—O3W—H3WB	100.3	C26—C25—H25A	120.4
H3WA—O3W—H3WB	136.0	C25—C26—C27	120.0 (2)
C22—O4—Mn2	136.17 (15)	C25—C26—H26A	120.0
Mn2—O4W—H4WA	93.6	C27—C26—H26A	120.0
Mn2—O4W—H4WB	121.8	N3—C27—C26	120.9 (2)
H4WA—O4W—H4WB	102.4	N3—C27—C28	116.1 (2)
C43—O5—Mn2	124.04 (16)	C26—C27—C28	123.0 (2)
H5WA—O5W—H5WB	83.4	N4—C28—C29	121.7 (2)
H6WA—O6W—H6WB	106.2	N4—C28—C27	115.7 (2)
C44—O8—Mn1^i^	132.04 (14)	C29—C28—C27	122.6 (2)
C1—N1—C5	117.9 (2)	C28—C29—C30	119.4 (3)
C1—N1—Mn1	124.66 (18)	C28—C29—H29A	120.3
C5—N1—Mn1	117.44 (15)	C30—C29—H29A	120.3
C10—N2—C6	117.5 (2)	C31—C30—C29	119.1 (3)
C10—N2—Mn1	124.08 (17)	C31—C30—H30A	120.4
C6—N2—Mn1	118.38 (16)	C29—C30—H30A	120.4
C23—N3—C27	118.2 (2)	C30—C31—C32	118.9 (3)
C23—N3—Mn2	123.94 (16)	C30—C31—H31A	120.5
C27—N3—Mn2	117.85 (15)	C32—C31—H31A	120.5
C32—N4—C28	118.0 (2)	N4—C32—C31	122.8 (3)
C32—N4—Mn2	124.01 (17)	N4—C32—H32A	118.6
C28—N4—Mn2	117.84 (16)	C31—C32—H32A	118.6
N1—C1—C2	123.2 (3)	C34—C33—C41	119.8 (3)
N1—C1—H1A	118.4	C34—C33—C43	114.2 (3)
C2—C1—H1A	118.4	C41—C33—C43	125.9 (2)
C3—C2—C1	118.8 (3)	C33—C34—C35	122.4 (3)
C3—C2—H2A	120.6	C33—C34—H34A	118.8
C1—C2—H2A	120.6	C35—C34—H34A	118.8
C2—C3—C4	119.4 (3)	C36—C35—C34	119.2 (3)
C2—C3—H3A	120.3	C36—C35—H35A	120.4
C4—C3—H3A	120.3	C34—C35—H35A	120.4
C3—C4—C5	119.2 (3)	C35—C36—C42	122.0 (3)
C3—C4—H4A	120.4	C35—C36—H36A	119.0
C5—C4—H4A	120.4	C42—C36—H36A	119.0
N1—C5—C4	121.6 (2)	C38—C37—C42	121.9 (3)
N1—C5—C6	116.4 (2)	C38—C37—H37A	119.0
C4—C5—C6	122.0 (2)	C42—C37—H37A	119.0
N2—C6—C7	121.9 (2)	C37—C38—C39	119.0 (3)
N2—C6—C5	115.8 (2)	C37—C38—H38A	120.5
C7—C6—C5	122.3 (2)	C39—C38—H38A	120.5
C6—C7—C8	119.7 (3)	C40—C39—C38	122.2 (3)
C6—C7—H7A	120.2	C40—C39—H39A	118.9
C8—C7—H7A	120.2	C38—C39—H39A	118.9
C9—C8—C7	118.8 (3)	C39—C40—C41	120.5 (3)
C9—C8—H8A	120.6	C39—C40—C44	115.5 (3)
C7—C8—H8A	120.6	C41—C40—C44	123.4 (2)
C8—C9—C10	118.8 (3)	C33—C41—C40	126.5 (2)
C8—C9—H9A	120.6	C33—C41—C42	117.1 (3)
C10—C9—H9A	120.6	C40—C41—C42	116.4 (3)
N2—C10—C9	123.4 (3)	C36—C42—C37	120.8 (3)
N2—C10—H10A	118.3	C36—C42—C41	119.5 (3)
C9—C10—H10A	118.3	C37—C42—C41	119.7 (3)
C12—C11—C19	119.3 (2)	O6—C43—O5	124.7 (2)
C12—C11—C21	114.2 (2)	O6—C43—C33	116.8 (2)
C19—C11—C21	126.2 (2)	O5—C43—C33	118.1 (2)
C11—C12—C13	123.0 (3)	O7—C44—O8	123.1 (2)
C11—C12—H12A	118.5	O7—C44—C40	119.8 (2)
C13—C12—H12A	118.5	O8—C44—C40	117.0 (2)
			
O2W—Mn1—O1—C21	64.86 (19)	C17—C18—C19—C20	4.1 (4)
O8^i^—Mn1—O1—C21	44.9 (3)	C22—C18—C19—C20	−169.9 (2)
O1W—Mn1—O1—C21	−32.36 (19)	C12—C11—C19—C18	178.5 (3)
N2—Mn1—O1—C21	−120.34 (19)	C21—C11—C19—C18	4.3 (4)
N1—Mn1—O1—C21	167.10 (19)	C12—C11—C19—C20	−0.7 (4)
O3W—Mn2—O4—C22	168.7 (2)	C21—C11—C19—C20	−175.0 (2)
O5—Mn2—O4—C22	125.1 (3)	C13—C14—C20—C15	−177.8 (3)
O4W—Mn2—O4—C22	67.2 (2)	C13—C14—C20—C19	0.0 (5)
N3—Mn2—O4—C22	−23.4 (2)	C16—C15—C20—C14	175.1 (3)
N4—Mn2—O4—C22	−96.1 (2)	C16—C15—C20—C19	−2.7 (4)
O4—Mn2—O5—C43	−23.0 (4)	C18—C19—C20—C14	−178.3 (2)
O3W—Mn2—O5—C43	−66.42 (18)	C11—C19—C20—C14	1.0 (4)
O4W—Mn2—O5—C43	34.68 (18)	C18—C19—C20—C15	−0.5 (4)
N3—Mn2—O5—C43	125.48 (18)	C11—C19—C20—C15	178.8 (2)
N4—Mn2—O5—C43	−161.66 (18)	Mn1—O1—C21—O2	4.7 (4)
O1—Mn1—N1—C1	−89.05 (19)	Mn1—O1—C21—C11	178.84 (15)
O2W—Mn1—N1—C1	−4.3 (2)	C12—C11—C21—O2	56.0 (3)
O8^i^—Mn1—N1—C1	75.76 (19)	C19—C11—C21—O2	−129.5 (3)
O1W—Mn1—N1—C1	158.48 (19)	C12—C11—C21—O1	−118.6 (3)
N2—Mn1—N1—C1	177.0 (2)	C19—C11—C21—O1	55.8 (3)
O1—Mn1—N1—C5	93.41 (16)	Mn2—O4—C22—O3	−105.4 (3)
O2W—Mn1—N1—C5	178.20 (15)	Mn2—O4—C22—C18	70.1 (3)
O8^i^—Mn1—N1—C5	−101.78 (16)	C17—C18—C22—O3	47.9 (3)
O1W—Mn1—N1—C5	−19.1 (3)	C19—C18—C22—O3	−137.7 (3)
N2—Mn1—N1—C5	−0.50 (15)	C17—C18—C22—O4	−127.8 (2)
O1—Mn1—N2—C10	82.40 (19)	C19—C18—C22—O4	46.5 (3)
O8^i^—Mn1—N2—C10	−92.99 (19)	C27—N3—C23—C24	−0.1 (4)
O1W—Mn1—N2—C10	−7.17 (19)	Mn2—N3—C23—C24	−177.8 (2)
N1—Mn1—N2—C10	179.4 (2)	N3—C23—C24—C25	−0.6 (4)
O1—Mn1—N2—C6	−95.01 (16)	C23—C24—C25—C26	0.5 (4)
O8^i^—Mn1—N2—C6	89.60 (16)	C24—C25—C26—C27	0.3 (4)
O1W—Mn1—N2—C6	175.42 (16)	C23—N3—C27—C26	1.0 (4)
N1—Mn1—N2—C6	2.01 (15)	Mn2—N3—C27—C26	178.81 (18)
O4—Mn2—N3—C23	87.9 (2)	C23—N3—C27—C28	−179.7 (2)
O3W—Mn2—N3—C23	173.8 (3)	Mn2—N3—C27—C28	−1.9 (3)
O5—Mn2—N3—C23	−84.6 (2)	C25—C26—C27—N3	−1.1 (4)
O4W—Mn2—N3—C23	2.9 (2)	C25—C26—C27—C28	179.6 (2)
N4—Mn2—N3—C23	−179.3 (2)	C32—N4—C28—C29	0.2 (4)
O4—Mn2—N3—C27	−89.76 (17)	Mn2—N4—C28—C29	−176.44 (19)
O3W—Mn2—N3—C27	−3.9 (4)	C32—N4—C28—C27	−179.0 (2)
O5—Mn2—N3—C27	97.70 (17)	Mn2—N4—C28—C27	4.4 (3)
O4W—Mn2—N3—C27	−174.78 (17)	N3—C27—C28—N4	−1.7 (3)
N4—Mn2—N3—C27	3.01 (16)	C26—C27—C28—N4	177.6 (2)
O4—Mn2—N4—C32	−85.9 (2)	N3—C27—C28—C29	179.2 (2)
O3W—Mn2—N4—C32	−1.8 (2)	C26—C27—C28—C29	−1.5 (4)
O5—Mn2—N4—C32	84.7 (2)	N4—C28—C29—C30	−0.6 (4)
O4W—Mn2—N4—C32	−172.7 (2)	C27—C28—C29—C30	178.5 (3)
N3—Mn2—N4—C32	179.6 (2)	C28—C29—C30—C31	0.1 (4)
O4—Mn2—N4—C28	90.49 (17)	C29—C30—C31—C32	0.7 (4)
O3W—Mn2—N4—C28	174.57 (17)	C28—N4—C32—C31	0.7 (4)
O5—Mn2—N4—C28	−98.93 (17)	Mn2—N4—C32—C31	177.1 (2)
O4W—Mn2—N4—C28	3.7 (4)	C30—C31—C32—N4	−1.1 (4)
N3—Mn2—N4—C28	−3.97 (16)	C41—C33—C34—C35	2.7 (4)
C5—N1—C1—C2	−0.4 (4)	C43—C33—C34—C35	−174.6 (2)
Mn1—N1—C1—C2	−177.9 (2)	C33—C34—C35—C36	−0.7 (4)
N1—C1—C2—C3	−0.6 (4)	C34—C35—C36—C42	0.1 (5)
C1—C2—C3—C4	1.0 (4)	C42—C37—C38—C39	−3.7 (5)
C2—C3—C4—C5	−0.5 (4)	C37—C38—C39—C40	0.7 (4)
C1—N1—C5—C4	0.9 (3)	C38—C39—C40—C41	4.3 (4)
Mn1—N1—C5—C4	178.60 (18)	C38—C39—C40—C44	−167.2 (2)
C1—N1—C5—C6	−178.6 (2)	C34—C33—C41—C40	176.1 (2)
Mn1—N1—C5—C6	−0.9 (2)	C43—C33—C41—C40	−7.0 (4)
C3—C4—C5—N1	−0.5 (4)	C34—C33—C41—C42	−3.8 (3)
C3—C4—C5—C6	179.0 (2)	C43—C33—C41—C42	173.1 (2)
C10—N2—C6—C7	−0.7 (3)	C39—C40—C41—C33	174.2 (2)
Mn1—N2—C6—C7	176.86 (17)	C44—C40—C41—C33	−15.1 (4)
C10—N2—C6—C5	179.3 (2)	C39—C40—C41—C42	−6.0 (3)
Mn1—N2—C6—C5	−3.1 (2)	C44—C40—C41—C42	164.8 (2)
N1—C5—C6—N2	2.6 (3)	C35—C36—C42—C37	178.9 (3)
C4—C5—C6—N2	−176.8 (2)	C35—C36—C42—C41	−1.4 (4)
N1—C5—C6—C7	−177.4 (2)	C38—C37—C42—C36	−178.5 (3)
C4—C5—C6—C7	3.2 (3)	C38—C37—C42—C41	1.7 (4)
N2—C6—C7—C8	0.5 (4)	C33—C41—C42—C36	3.2 (3)
C5—C6—C7—C8	−179.5 (2)	C40—C41—C42—C36	−176.7 (2)
C6—C7—C8—C9	−0.1 (4)	C33—C41—C42—C37	−177.1 (2)
C7—C8—C9—C10	−0.1 (4)	C40—C41—C42—C37	3.1 (3)
C6—N2—C10—C9	0.6 (4)	Mn2—O5—C43—O6	−10.1 (3)
Mn1—N2—C10—C9	−176.84 (19)	Mn2—O5—C43—C33	176.67 (15)
C8—C9—C10—N2	−0.2 (4)	C34—C33—C43—O6	−48.6 (3)
C19—C11—C12—C13	−0.6 (4)	C41—C33—C43—O6	134.4 (2)
C21—C11—C12—C13	174.3 (3)	C34—C33—C43—O5	125.2 (2)
C11—C12—C13—C14	1.7 (5)	C41—C33—C43—O5	−51.8 (3)
C12—C13—C14—C20	−1.3 (5)	Mn1^i^—O8—C44—O7	107.6 (3)
C20—C15—C16—C17	2.3 (5)	Mn1^i^—O8—C44—C40	−68.3 (3)
C15—C16—C17—C18	1.4 (5)	C39—C40—C44—O7	−41.9 (3)
C16—C17—C18—C19	−4.7 (4)	C41—C40—C44—O7	146.9 (2)
C16—C17—C18—C22	169.9 (2)	C39—C40—C44—O8	134.2 (2)
C17—C18—C19—C11	−175.1 (2)	C41—C40—C44—O8	−37.0 (3)
C22—C18—C19—C11	10.8 (4)		

Symmetry codes: (i) −*x*+1, −*y*+1, −*z*+1.

### Hydrogen-bond geometry (Å, °)

**Table:**

*D*—H···*A*	*D*—H	H···*A*	*D*···*A*	*D*—H···*A*
O1W—H1WA···O2	0.85	2.00	2.690 (2)	139
O1W—H1WB···O6^i^	0.84	2.01	2.776 (2)	151
O2W—H2WA···O4	0.83	1.90	2.712 (2)	166
O2W—H2WB···O5W	0.83	1.90	2.716 (3)	169
O3W—H3WA···O8	0.84	1.92	2.742 (2)	167
O3W—H3WB···O2W	0.84	2.24	3.083 (3)	180
O4W—H4WA···O6	0.87	1.78	2.622 (2)	161
O4W—H4WB···O6W	0.83	2.03	2.853 (3)	170
O5W—H5WA···O7^i^	0.97	2.16	2.807 (3)	123
O5W—H5WB···O6W	0.94	2.07	2.930 (3)	150
O6W—H6WA···O7^ii^	0.84	1.99	2.798 (3)	164
O6W—H6WB···O3	0.81	1.95	2.752 (3)	171

Symmetry codes: (i) −*x*+1, −*y*+1, −*z*+1; (ii) *x*−1, *y*, *z*.
